# Hydrocephalus after Subarachnoid Hemorrhage: Pathophysiology, Diagnosis, and Treatment

**DOI:** 10.1155/2017/8584753

**Published:** 2017-03-08

**Authors:** Sheng Chen, Jinqi Luo, Cesar Reis, Anatol Manaenko, Jianmin Zhang

**Affiliations:** ^1^Department of Neurosurgery, Second Affiliated Hospital, School of Medicine, Zhejiang University, Hangzhou, Zhejiang, China; ^2^Brain Research Institute, Zhejiang University, Hangzhou, China; ^3^Department of Physiology and Pharmacology, Loma Linda University, Loma Linda, CA, USA; ^4^Department of Preventive Medicine, Loma Linda University, Loma Linda, CA, USA; ^5^Department of Neurology, University of Erlangen-Nuremberg, Erlangen, Germany; ^6^Collaborative Innovation Center for Brain Science, Zhejiang University, Hangzhou, Zhejiang, China

## Abstract

Hydrocephalus (HCP) is a common complication in patients with subarachnoid hemorrhage. In this review, we summarize the advanced research on HCP and discuss the understanding of the molecular originators of HCP and the development of diagnoses and remedies of HCP after SAH. It has been reported that inflammation, apoptosis, autophagy, and oxidative stress are the important causes of HCP, and well-known molecules including transforming growth factor, matrix metalloproteinases, and iron terminally lead to fibrosis and blockage of HCP. Potential medicines for HCP are still in preclinical status, and surgery is the most prevalent and efficient therapy, despite respective risks of different surgical methods, including lamina terminalis fenestration, ventricle-peritoneal shunting, and lumbar-peritoneal shunting. HCP remains an ailment that cannot be ignored and even with various solutions the medical community is still trying to understand and settle why and how it develops and accordingly improve the prognosis of these patients with HCP.

## 1. Introduction

Hydrocephalus (HCP) is a serious and common complication in the clinical course of subarachnoid hemorrhage (SAH), which continues to be vague until now. According to various background and clinical circumstances, wide range of incidence of HCP in SAH patients from 6 to 67% has been reported; in most recent studies this percentage is about 20%~30%.

HCP occurs in about one fifth of patients in the early course (acute in the first 3 days or subacute in the 4–14 days) of SAH, while chronic hydrocephalus happens in 10%–20% of patients later in the course of SAH (after 2 weeks). Regardless of the occurring period, HCP impairs patient's neurologic function and leads to deterioration of functional outcomes, especially with intraventricular hemorrhage (IVH), even if the primary SAH has been treated [1]. On the contrary, better outcomes occur if SAH is recognized early and treated.

Despite not having satisfactory preventive treatments, there have been several therapeutic methods developed to deal with hydrocephalus or to minimize the necessity of permanent shunts. Intraoperatively, lamina terminalis fenestration (LTF) with thorough lavage of blood clots out of ventricles and cisterns is carried out in order to reconstruct the normal flow course of cerebrospinal fluid (CSF) and also to eliminate the impairments by blood clots and its by-products. Postoperatively, temporary intraventricular or lumbar drainage is a technique used to transfer CSF reabsorption. For patients without intraventricular catheters or lumbar drains but with persistent symptoms, serial lumbar punctures are necessary. Despite these efforts to prevent the occurrence, a considerable number of patients are in need of a perennial shunt for CSF.

In this review we summarize the research of SAH-induced HCP and discuss the etiology, diagnosis, and treatment. With this field advancing thanks to the efforts of many researchers, questions and problems on treatment and prevention remain to be solved and applied to clinical practice.

## 2. Etiology

About one third of patients admitted with SAH have permanent CSF diversion. A large-scale meta-analysis reported that shunt-dependent HCP accounts for a proportion of 17.4% [2]. Patients with acute course, in-hospital complications, IVH, poor admission status, rehemorrhage, location of ruptured aneurysm, and age ≥ 60 reported a higher risk of shunt dependency [3–5].

**Figure 1:**
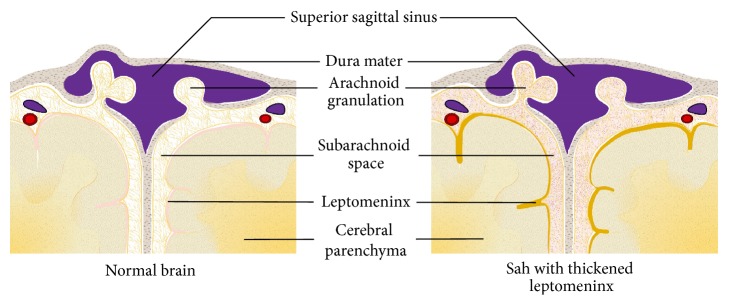
After SAH, the subarachnoid space is filled with blood cells and products. Leptomeninx is detected thickened with hemosiderin deposits, which has also been confirmed histologically.

**Figure 2:**
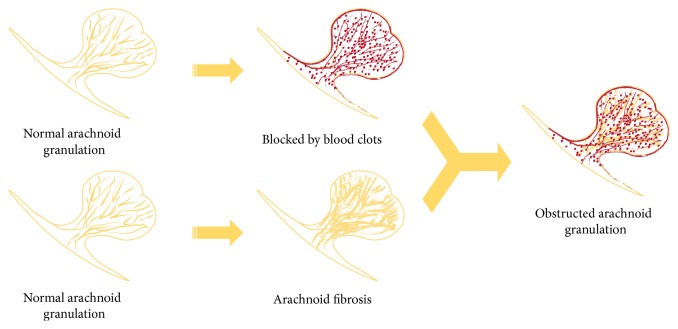
This picture shows the major pathological mechanisms in arachnoid granulations; the upper ones demonstrate the blood clots and corresponding products blocking the outflow tract of CSF and the inferior ones show the fibrosis of arachnoid membrane meanwhile.

Achievements and progress in studying hydrocephaly inevitably fall short of elucidating the entire mechanism of HCP after SAH. The theories mentioned hereinbefore meet the questions of researchers approximately through damage to arachnoid granulations (AGs) as well as to brain tissue. Mechanisms seem to be interweaving among the pathogenesis of acute and chronic HCP. It is generally accepted that the inflammatory reaction (either chronic or acute) and the ensuing fibrosis process impede fluent CSF flow outward to sinus, terminally from AGs. Beside the proliferation of leptomeningeal cells (Figure 1), studies at present primarily target the pathological obstruction of AGs, including the mechanical blockage and fibrosis of AGs (Figure 2). Researchers have been long working on attenuating this pathogenesis to deal with HCP [6, 7].

**Figure 3:**
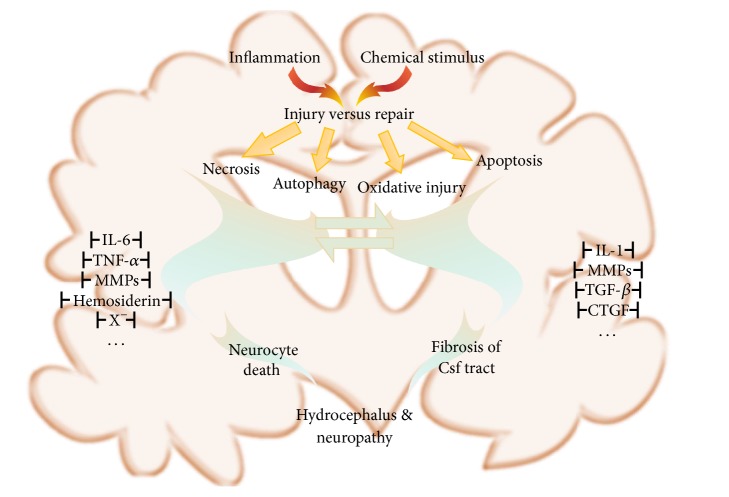
This picture shows some broadly verified molecules or pathways that are involved in the pathophysiogenesis of hydrocephalus caused by SAH.

Researchers mostly focus on the pathophysiology of brain injury after SAH, and prevalent theories include inflammation, apoptosis, autophagy, and oxidative stress (Figure 3). Vasospasm of choroidal artery probably originates HCP through stenosing the aqueduct and impairing ependymal cells after SAH [8]. Devascularization of brain parenchyma likely results from sequential vasospasm of SAH and is confirmed to induce the proliferation of neural stem cells directed by glia cells [9]. Gliocytes, different from other organs of the body, play the destructive and curative roles and release plenty of cytokines when the brain suffers various lesions [10]. Matrix metalloproteinases are believed to be crucial and versatile participants in breaking down blood-brain barrier (BBB) [11], and the tissue inhibitors of matrix metalloproteinases have been verified to share the homologous protective effects in vasospasm after SAH for BBB integrity in apoplectic patients [12]. In addition, researchers found that the vegetative nervous system plays an auxiliary role in the inflammatory response and may contribute to the breakdown of BBB, which consists of glia cell both structurally and functionally [13]. Vascular endothelial growth factor protein levels rise and restrict the growth of abnormal blood vessels [14]. Subsequently, the hypersecretion of CSF triggers or exacerbates its circulatory disorder and eventually leads to HCP.

Acute HCP contributes to the causes of early brain injuries [15], usually thought as the noncommunicating (or obstructive) type, and is largely attributed to the mass effect or blood clots within the ventricles and aqueduct, preventing CSF flow out of the cranial vault. In addition, inflammation is believed to be the crucial biomolecular mechanism that induces acute HCP through disruption of BBB [16]. Nevertheless, recent research illustrated radiologically similar performances between acute and chronic HCP, indicating partially similar pathogenesis. Phase-contrast MRI demonstrated that chronic HCP turns out to be of communicating form; however, some of these individuals still develop acute HCP after SAH despite the absence of IVH or blood clot in the ventricles [17, 18]. Parallel parameters of CSF flow found in their studies also indicated that obstruction might not be sole initiator of acute HCP. Additionally, Kanat et al. postulated that blood clots play the initial role in triggering hypersecretion of CSF and fibrosis of arachnoid granulations, leading to long-term communicating HCP rather than merely aqueduct obstruction or stenosis [19]. Whether it is communicating, obstructive, or a pathophysiological hybrid, it may directly affect the treatment decision and corresponding prognosis of these patients. Despite many discoveries and advances, more evidence is needed to uncover and explain the etiology of acute HCP following hemorrhage.

Conversely, a considerable number of patients with chronic HCP have no increased intracranial pressure (ICP) and with abundant evidence emerging in the pathway of fibrosis, there is a general consensus that chronic HCP is of “communicating” type, attributed to the fibrosis and adhesions of the leptomeningeal and arachnoid granulations. Blood products and transforming growth factor have long been postulated to play important roles in the pathophysiological processes after SAH, including chronic HCP. Intraventricularly injected iron (ferrous chloride or ferric chloride) or lysed red blood cells can similarly lead to HCP in rats [20]. In addition, Strahle et al. also detected cell deaths in neonatal rat model through pathological sections [21], which has testified the very critical effects in the mechanisms. Furthermore, necrosis of brain cells and disruption of BBB induced by iron are also depicted in rats [22], which makes this postulation more eloquent. Given all the previous research, preclinical research is supportive of the idea that oxidation accounts for the precise mechanisms of pathogenesis induced by iron [23], initially termed “ferroptosis” [24]. But more evidence is needed to further unravel the proceedings and connections between “ferroptosis” and HCP, and we are longing for a convincible clinical trial to testify whether removing the blood clot or subarachnoid blood lavage in the initial stage of SAH will have a definite positive outcome in these patients.

## 3. Diagnosis

**Figure 4:**
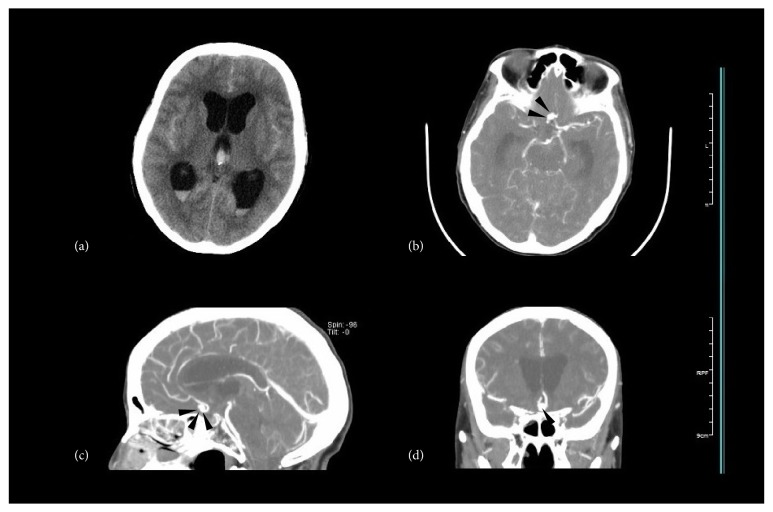
This picture shows a case of acute HCP induced by aneurysmal SAH, typically with an IVH. It happened as soon as the occurrence of SAH. (a) The CT scans above show widely hemorrhagic sulci and arachnoid cisterns with dilated lateral and third ventricles containing blood. (b), (c), and (d) Immediate CTA after admission locates the culprit aneurysm on the anterior communicating artery (marked by black arrows).

**Figure 5:**
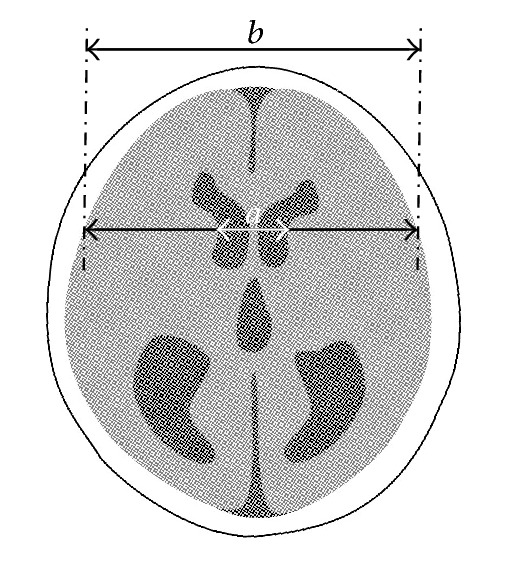
This picture simulates how to calculate the BCI, namely, the severity of HCP, the ratio. Segment “*a*” is the distance between caudate nuclei and “*b*” is at the same level the width of brain. The ratio “*a*/*b*” of respective group of age, that is, relative bilateral caudate index is also widely accepted among researchers.

Compared with detection of chronic HCP occurring during or after the course of SAH, it is more difficult to clinically diagnose acute HCP, which can be misleading or concealed by SAH accompanied with headache, nausea, or conscious disturbance. Since it involves ventricular dilation anatomically, its recognition is primarily based on radiographic techniques, especially CT scans (Figure 4). The bicaudate index (BCI) and relative bicaudate index (RBCI) (calculated, resp., in different age groups) have been commonly accepted and widely applied as the diagnostic measurements since the study of Gijn and colleagues in 1980s (as shown in Figure 5) [17, 25–28]. And peers draw a conclusion that if not detected promptly before RBCI > 1.6, the effort to launch a drainage surgery could be in vain because of unimproved outcomes [29]. Still, the form and shape of dilated ventricles in patients differ a lot, and the authors suppose it is more accurate to measure the volume of ventricles and calculate the dilation rate [30].

Advances in radiological imaging and studies and useful methods such as diffusion tensor image (DTI) [31] and diffusional kurtosis image (DKI) are utilized [32], but CT is still the fastest and most efficient diagnostic one for HCP. Moreover, MRI gives much more details regarding whether or not and how brain parenchyma is damaged by ventricular dilation. What is more, we can observe precisely the morphology of the aqueduct and dynamics of CSF and subsequently know if it is blocked or stenosed [17, 18]. These advanced examinations provide more details in patients than CT scans, which are likely to facilitate unveiling the etiology and pathogenesis of HCP. One study demonstrated both the altered microstructure and water molecule movement within neural axons and intra- or extracellular space in patients with idiopathic normal pressure hydrocephalus (iNPH) by DTI and DKI [33]. These findings may be useful in evaluating the brain damage after SAH and HCP [34].

## 4. Predictive Factors

A considerable number of patients are exposed to the risk of shunt-dependent HCP after SAH. Earlier diversion of CSF results in less damage to brain parenchyma. Difficulties exist in deciding whether to intermittently launch drainage or perform surgery to divert CSF secreted beyond absorption. It is important and beneficial to predict shunt-dependence beyond its clinical performances [35]. Patients with acute course of HCP, in-hospital complications, IVH, high Hunt and Hess Scale score (or low initial Glasgow Coma Scale or high Fisher score), rehemorrhage, posterior circulation location of ruptured aneurysm, and age ≥ 60 have been reported to be at a higher risk of shunt-dependency [3–5]. Other research reported similarly higher risk of HCP with posterior circulation aneurysm, IVH, greater hemorrhage volume, and older age [4, 5, 28, 36]. Dependency on factors like economy, medical development, and methods to cope with ruptured aneurysms also leads to deferent incidences of shunt-dependent HCP [5].

In addition, some researchers attempt to find a precise and measurable way to foresee the perennial shunting necessity. In the study of Hoh et al., symptomatic aneurysms are found likely larger and more likely to cause obstructive hydrocephalus, which may need a drainage operation [37]. Yamada et al. in 2012 introduced a discriminant function relevant to determining the need for VPS after SAH [38]. The sensitivity and specificity were at 85.3% and 87.2%, respectively, which are high enough for predicting shunt-independence. This is favorable to earlier surgical performance and prevents damage caused by ventriculomegaly. More evidence and cases are needed to develop a function model more clinically applicable and usable.

## 5. Treatment

### 5.1. Medical Treatments

Common medical treatments for HCP mainly include acetazolamide and mannitol. It has been testified by perennial clinical practice that medication does not reduce the possibility of subsequent surgical drainage, with extra side effects. It is now applied in hopes of putting off shunt-placement surgery and preoperative preparation.

Along with the gradual disclosure of mechanisms in HCP in recent years, some experimental agents are found to be potentially effective in improving the outcomes of patients [7, 39]. Minocycline is reported to be effective in reducing the gliosis and delaying the development of HCP in rat model [40]. And decorin may be beneficial for the long-term of HCP [7]. On the other hand, in neonatal rats with germinal matrix hemorrhage, deferoxamine attenuates long-term complications including posthemorrhagic dilation of ventricles [41].

SAH shares similar mechanisms with intracerebral hemorrhage and contributes to detrimental processes that include HCP and brain apoptosis. In this regard, they might have similar treatments. Trichostatin A (TSA), histone deacetylase inhibitor which enhances autophagy, contributes to alleviation of neuronal apoptosis, improvement of neurological function, and attenuation of brain injury following SAH [42], potentially leading to slighter fibrosis of meninx and better outcomes of patients with HCP.

### 5.2. Surgical Treatments

Despite a considerably high incidence of complications, about 50%, shunt failures within 1 year, about 30%, and a number of patients in need of a secondary surgery to revise the catheter, surgery is still the preferred treatment for HCP. The aim of surgical treatment is to improve the neurofunction by CSF flow diversion rather than restore the original cerebral structure. Surgical protocol differs depending on the type of hydrocephalic lesion and the conditions of individual patients. The optimal time for surgical treatments remains controversial. Three predominant surgical methods for HCP are compared with each other in Table 1.

#### 5.2.1. Lamina Terminalis Fenestration (LTF)

Reported to have less complications and being favorable in reducing shunt-dependent occurrence [43–45], surgeons incline to launch LTF during surgical operation for acute SAH after lavage of blood clots in the subarachnoid space to avoid posthemorrhagic obstruction of CSF flow. However, some other researchers questioned the efficacy of LTF to cut down shunt-dependence of patients [46]. As mentioned in our passage about the pathogenesis of acute HCP, LTF does not terminate or delay the fibrotic process of leptomeninges and arachnoid granulations, hence possibly improving CSF dynamics. Authors remain suspicious of its effects and long-term outcomes, mainly the shunt-dependent incidence on acute patients, especially those who suffer from communicating HCP without early diagnostic evidence.

#### 5.2.2. Ventricle-Peritoneal Shunting (VPS)

VPS is currently the most widely applied surgical method to deal with HCP. According to a systematic review involving 41,789 patients with aneurysmal SAH in 66 published studies, the overall VPS insertion rate was 12.7% [47]; 31.2% patients required a VPS for acute HCP after aneurysmal SAH, regardless of whether it was after endovascular or surgical treatment [48].

However, even though it is the most commonly applicative surgical protocol, VPS bears an inevitable high risk of complications and failures. A 10-year follow-up among 14,455 individuals who underwent VPS showed 32% had cumulative complications at 5 years [49]. Another clinical study exhibited 51.9% patients accepting VPS requiring shunt revision(s) [50]. Occurrence of complications mostly attributes to the implantation of the catheter and communication between ventricles, cisterns, and enterocoelia. The way in which surgeons implant the tube and how they set the parameters of the CSF sluice play a significant role in determining the outcomes of patients.

#### 5.2.3. Lumbar-Peritoneal Shunting (LPS)

LPS is usually performed as a supplementary solution for patients who suffer from communicating HCP that are not suitable for VPS. Compared with VPS, LPS involves a much shorter catheter, consequently slighter complications such as excessive shunt, intracranial pressure fluctuation, slit ventricles, and infection. On the other hand, LPS occupies a more narrow scope of application for curing HCP.

## 6. Conclusion

HCP occurrence after SAH presents with various clinical characteristics and mysterious biomolecular mechanisms that are still not addressed. Even though some studies demonstrated the pathophysiology includes fibrosis and obstruction of arachnoid, corresponding risk factors, which are generalized by predecessors, still contribute limitedly to avoiding HCP. Several surgical methods including LTF, VPS, and LPS are available but deficient in avoiding or treating hydrocephalus. However, the medical research community continues to discover mechanisms involved and more efficient and beneficial treatments for patients.

## Figures and Tables

**Table 1:** Comparison among dominant treatment methods.

	Lamina terminalis fenestration (LTF)	Ventricle-peritoneal shunting (VPS)	Lumbar-peritoneal shunting (LPS)
Advantages	(1) Less injuries;		
	(2) no implanted materials and less related	(1) Higher availability;	
	complications;	(2) more beneficial outcome	
	(3) conform to normal CSF dynamics;		
	(4) milder fluctuation of ICP		

Indication	Preferred for obstructive HCP, especially for those with mesencephalic aqueduct obstructed	Communicating HCP; some obstructive patients	Only for communicating HCP ≥ 2-year-old

Common complications	CSF leakage, meningitis, bleeding, basal artery injury, hypothalamic damage, epilepsy	Device fault, infection, excessive shunt, intracranial hypotension, slit ventricles, subdural hematoma or hydrops, displacement, visceral injury, epilepsy	
